# The genome sequence of the Common Emerald, *Hemithea aestivaria *(Hübner, 1789)

**DOI:** 10.12688/wellcomeopenres.19542.1

**Published:** 2023-06-14

**Authors:** Douglas Boyes, Peter W.H. Holland, University of Oxford and Wytham Woods Genome Acquisition Lab, Darwin Tree of Life Barcoding collective, Wellcome Sanger Institute Tree of Life programme, Wellcome Sanger Institute Scientific Operations: DNA Pipelines collective, Tree of Life Core Informatics collective, Darwin Tree of Life Consortium

**Affiliations:** 1 UK Centre for Ecology & Hydrology, Wallingford, England, UK; 2 University of Oxford, Oxford, England, UK

**Keywords:** Hemithea aestivaria, Common Emerald, genome sequence, chromosomal, Lepidoptera

## Abstract

We present a genome assembly from an individual male *Hemithea aestivaria* (the Common Emerald; Arthropoda; Insecta; Lepidoptera; Geometridae). The genome sequence is 501.7 megabases in span. Most of the assembly is scaffolded into 31 chromosomal pseudomolecules, including the Z sex chromosome. The mitochondrial genome has also been assembled and is 17.05 kilobases in length. Gene annotation of this assembly on Ensembl identified 18,477 protein coding genes.

## Species taxonomy

Eukaryota; Metazoa; Eumetazoa; Bilateria; Protostomia; Ecdysozoa; Panarthropoda; Arthropoda; Mandibulata; Pancrustacea; Hexapoda; Insecta; Dicondylia; Pterygota; Neoptera; Endopterygota; Amphiesmenoptera; Lepidoptera; Glossata; Neolepidoptera; Heteroneura; Ditrysia; Obtectomera; Geometroidea; Geometridae; Geometrinae; *Hemithea; Hemithea aestivaria* (Hübner, 1789) (NCBI:txid572857).

## Background

The ‘emeralds’ are a group of over 2000 moth species, most of which have characteristic blue-green wings. Phylogenetic analysis using a small number of genes suggests the group is monophyletic and it is currently classified as a distinct subfamily Geometrinae within the family Geometridae ( Ban *et al.*, 2018; Sihvonen *et al.*, 2011). Of the 10–12 species found in Britain and Ireland, the Common Emerald, *Hemithea aestivaria*, is one of the most widespread and can be recognised by its dark green angular wings with black and white chequered fringes.

The geographic range of *H. aestivaria* spans much of Eurasia, from Portugal and Ireland to Japan and Korea ( GBIF Secretariat, 2022). In Britain, the moth is most common in the southern counties of England and has a northern limit in the south of Scotland ( Randle *et al.*, 2019). In Europe, the moth in univoltine with the adult flying in summer; the polyphagous larvae feed on low-growing herbaceous plants in autumn, and after overwintering eat the leaves of woody trees and bushes ( South, 1961). *H. aestivaria* is recorded as bivoltine in Japan ( Hausmann, 2001). The species is thought to have been introduced accidentally to North America: larvae were first recorded on fruit trees in British Columbia in 1973 before the species spread south to Oregon and Washington State where it is a minor pest of apple orchards ( Doǧanlar & Beirne, 1979; LaGasa, 1996; Looney *et al.*, 2016; Schmidt & Anctil, 2021). Since 2019, *H. aestivaria* has also been recorded on the east coast of Canada in Ontario, Québec and Nova Scotia ( Schmidt & Anctil, 2021).

The green colour of emerald moths has long intrigued entomologists due to its propensity to fade in living individuals and in dried museum specimens. It also a recognisably different shade to the green on other lepidopteran wings. The colour is conferred by a pigment located in granules within the wing scales; extractions using wings of *H. aestivaria* and other emerald moths has shown it to be a light-sensitive polar molecule, most likely bound to protein ( Cook, 1993; Cook *et al.*, 1994). The chemical structure of the pigment (named geoverdin) and the biochemical pathway for its production have not been determined.

The genome sequence of *Hemithea aestivaria* was determined as part of the Darwin Tree of Life project. The assembled genome sequence will facilitate research into the biochemistry underpinning pigment synthesis in insects, and contribute to the growing set of resources for studying lepidopteran ecology and evolution.

## Genome sequence report

**Figure 1.**
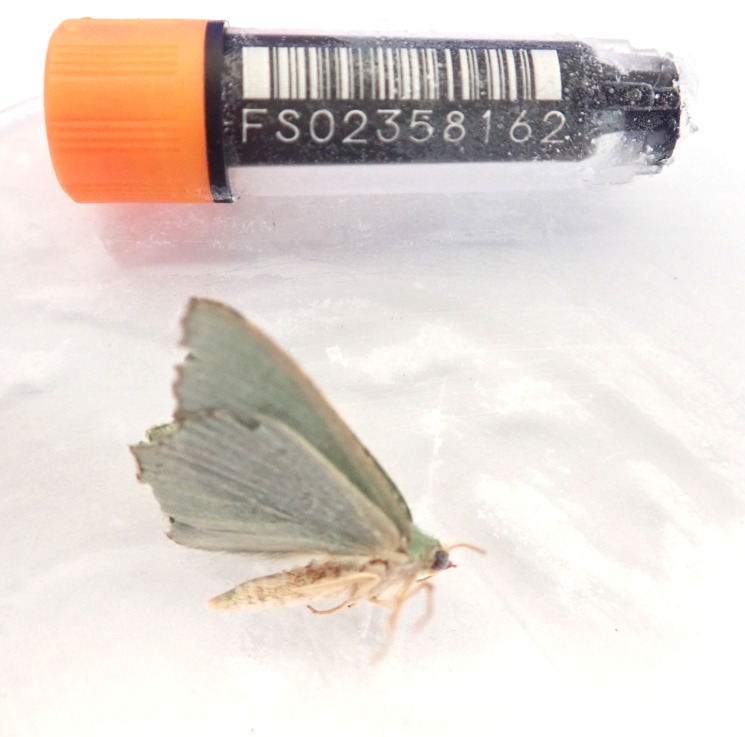
Photograph of the *Hemithea aestivaria* (ilHemAest2) specimen used for genome sequencing.

The genome was sequenced from one male *Hemithea aestivaria* ( Figure 1) collected from Wytham Woods, Oxfordshire (51.77, –1.34). A total of 36-fold coverage in Pacific Biosciences single-molecule HiFi long reads was generated. Primary assembly contigs were scaffolded with chromosome conformation Hi-C data. Manual assembly curation corrected 67 missing joins or mis-joins and removed 17 haplotypic duplications, reducing the assembly length by 0.64% and the scaffold number by 10.48%, and increasing the scaffold N50 by 0.48%.

**Figure 2.**
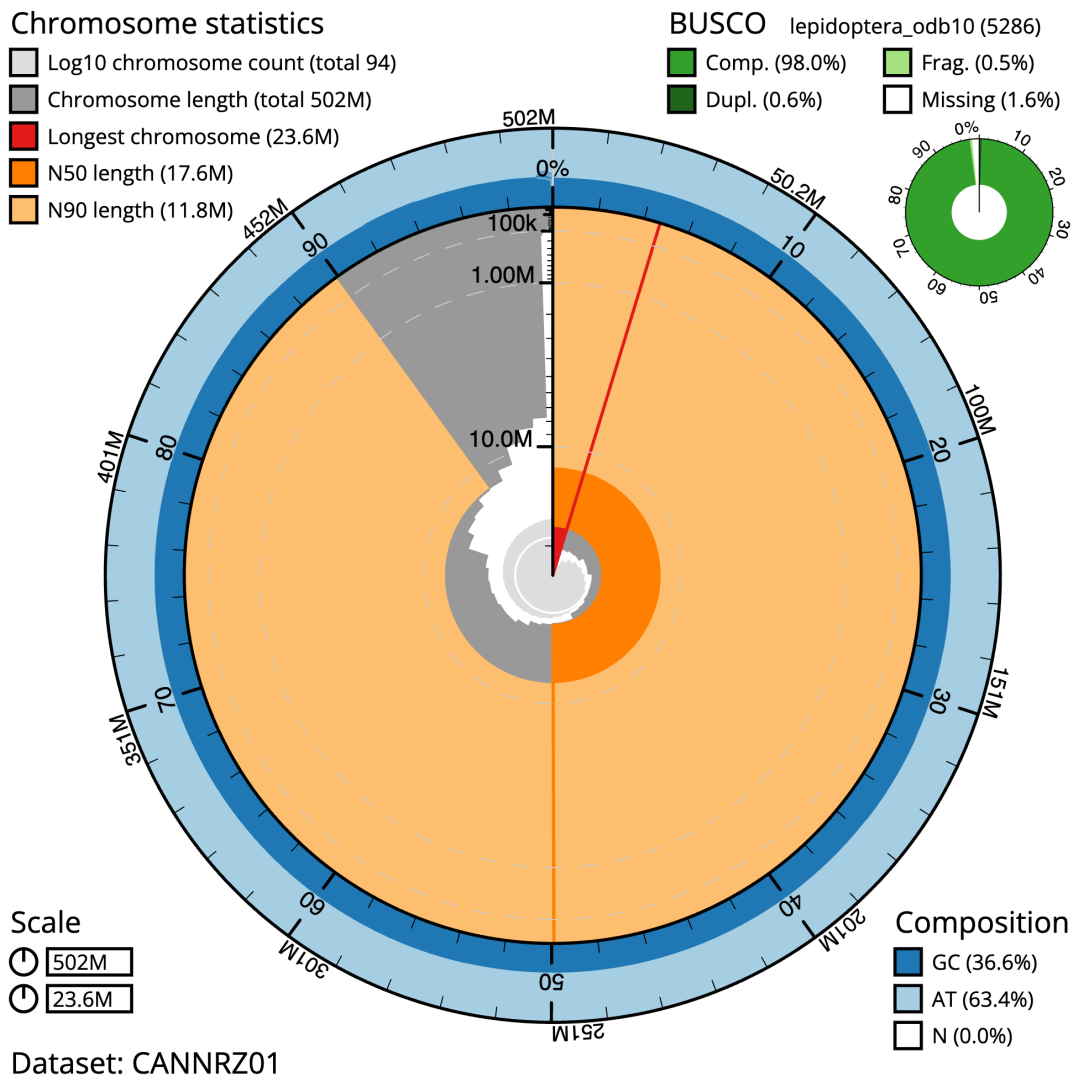
Genome assembly of *Hemithea aestivaria*, ilHemAest2.1: metrics. The BlobToolKit Snailplot shows N50 metrics and BUSCO gene completeness. The main plot is divided into 1,000 size-ordered bins around the circumference with each bin representing 0.1% of the 501,713,186 bp assembly. The distribution of scaffold lengths is shown in dark grey with the plot radius scaled to the longest scaffold present in the assembly (23,607,823 bp, shown in red). Orange and pale-orange arcs show the N50 and N90 scaffold lengths (17,632,203 and 11,810,528 bp), respectively. The pale grey spiral shows the cumulative scaffold count on a log scale with white scale lines showing successive orders of magnitude. The blue and pale-blue area around the outside of the plot shows the distribution of GC, AT and N percentages in the same bins as the inner plot. A summary of complete, fragmented, duplicated and missing BUSCO genes in the lepidoptera_odb10 set is shown in the top right. An interactive version of this figure is available at https://blobtoolkit.genomehubs.org/view/ilHemAest2.1/dataset/CANNRZ01/snail.

The final assembly has a total length of 501.7 Mb in 93 sequence scaffolds with a scaffold N50 of 17.6 Mb ( Table 1). Most (99.38%) of the assembly sequence was assigned to 31 chromosomal-level scaffolds, representing 30 autosomes and the Z sex chromosome. Chromosome-scale scaffolds confirmed by the Hi-C data are named in order of size ( Figure 2– Figure 5; Table 2). While not fully phased, the assembly deposited is of one haplotype. Contigs corresponding to the second haplotype have also been deposited. The mitochondrial genome was also assembled and can be found as a contig within the multifasta file of the genome submission.

**Table 1.** Genome data for *Hemithea aestivaria*, ilHemAest2.1.

Project accession data		
Assembly identifier	ilHemAest2.1	
Species	*Hemithea aestivaria*	
Specimen	ilHemAest2	
NCBI taxonomy ID	572857	
BioProject	PRJEB56491	
BioSample ID	SAMEA7701444	
Isolate information	ilHemAest2	
Assembly metrics *		*Benchmark*
Consensus quality (QV)	59.5	*≥ 50*
*k*-mer completeness	100%	*≥ 95%*
BUSCO **	C:98.0%[S:97.4%,D:0.6%], F:0.5%,M:1.6%,n:5,286	*C ≥ 95%*
Percentage of assembly mapped to chromosomes	99.38%	*≥ 95%*
Sex chromosomes	Z chromosome	*localised homologous pairs*
Organelles	Mitochondrial genome assembled	*complete single alleles*
Raw data accessions		
PacificBiosciences SEQUEL II	ERR10357397	
Hi-C Illumina	ERR10323148	
Genome assembly		
Assembly accession	GCA_947507615.1	
*Accession of alternate haplotype*	GCA_947461825.1	
Span (Mb)	501.7	
Number of contigs	377	
Contig N50 length (Mb)	2.6	
Number of scaffolds	93	
Scaffold N50 length (Mb)	17.6	
Longest scaffold (Mb)	23.6	
Genome annotation		
Number of protein-coding genes	18,477	
Number of gene transcripts	18,682	

* Assembly metric benchmarks are adapted from column VGP-2020 of “Table 1: Proposed standards and metrics for defining genome assembly quality” from ( Rhie *et al.*, 2021). ** BUSCO scores based on the lepidoptera_odb10 BUSCO set using v5.3.2. C = complete [S = single copy, D = duplicated], F = fragmented, M = missing, n = number of orthologues in comparison. A full set of BUSCO scores is available at https://blobtoolkit.genomehubs.org/view/ilHemAest2.1/dataset/CANNRZ01/busco.

**Figure 3.**
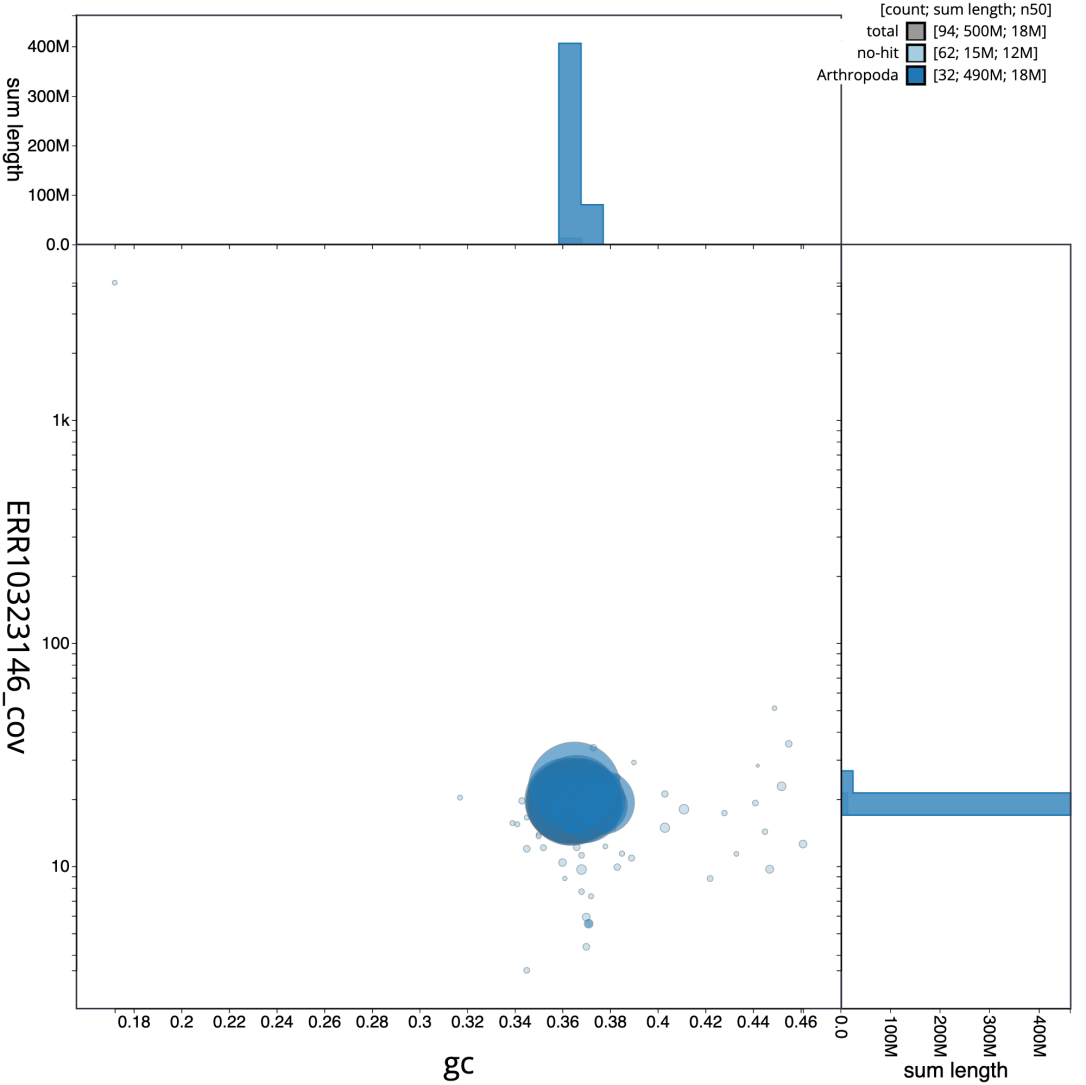
Genome assembly of *Hemithea aestivaria*, ilHemAest2.1: BlobToolKit GC-coverage plot. Scaffolds are coloured by phylum. Circles are sized in proportion to scaffold length. Histograms show the distribution of scaffold length sum along each axis. An interactive version of this figure is available at https://blobtoolkit.genomehubs.org/view/ilHemAest2.1/dataset/CANNRZ01/blob.

**Figure 4.**
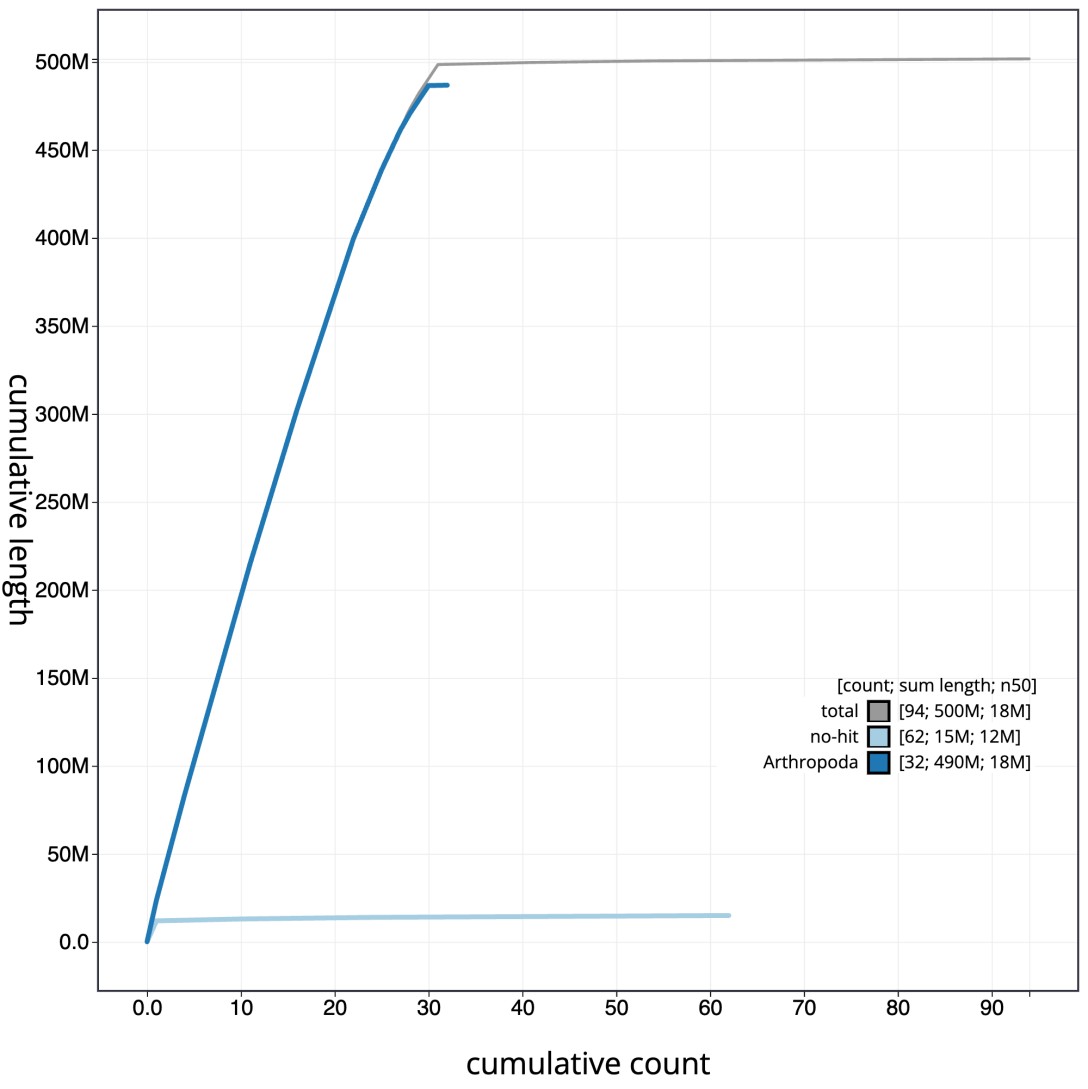
Genome assembly of *Hemithea aestivaria*, ilHemAest2.1: BlobToolKit cumulative sequence plot. The grey line shows cumulative length for all scaffolds. Coloured lines show cumulative lengths of scaffolds assigned to each phylum using the buscogenes taxrule. An interactive version of this figure is available at https://blobtoolkit.genomehubs.org/view/ilHemAest2.1/dataset/CANNRZ01/cumulative.

**Figure 5.**
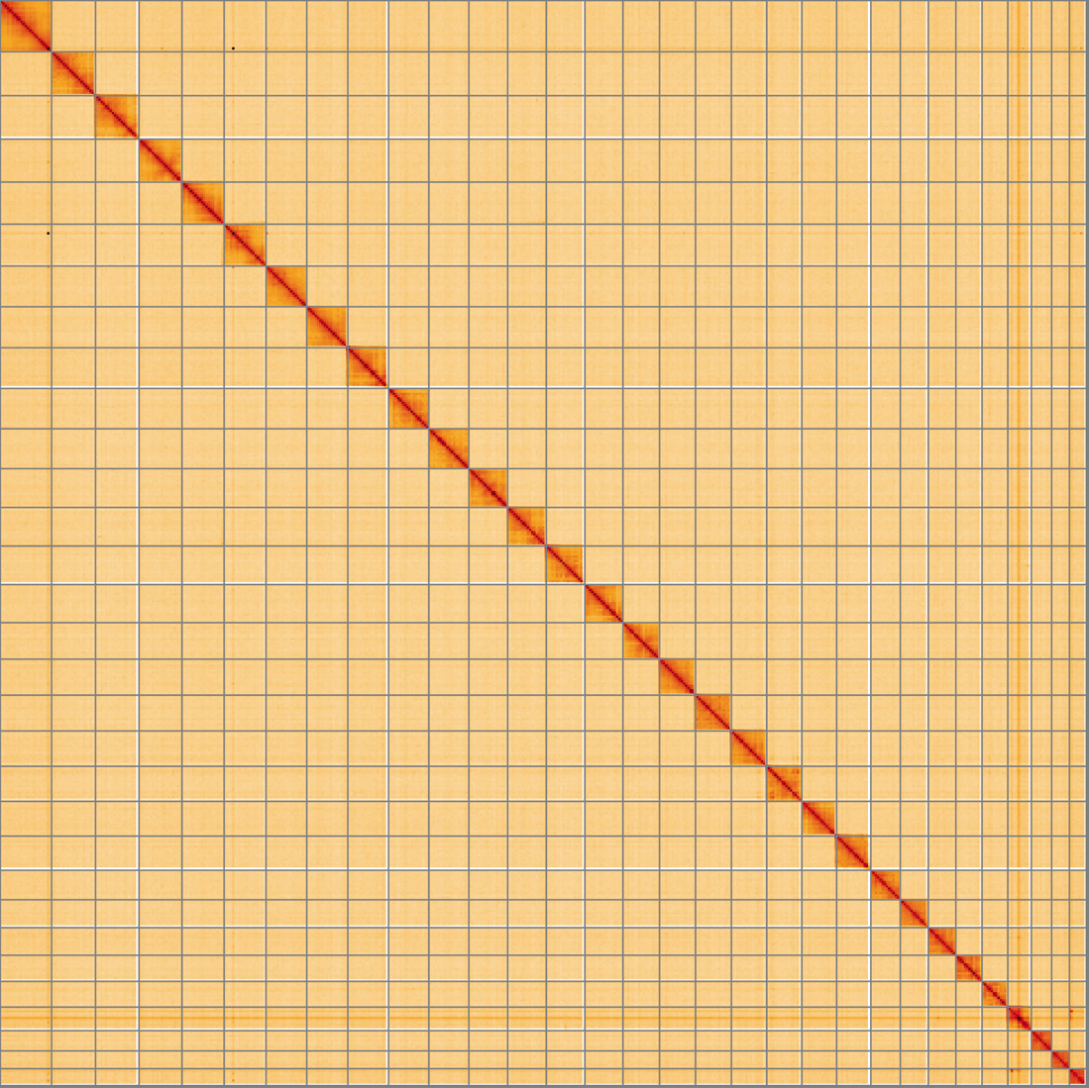
Genome assembly of *Hemithea aestivaria*, ilHemAest2.1: Hi-C contact map of the ilHemAest2.1 assembly, visualised using HiGlass. Chromosomes are shown in order of size from left to right and top to bottom. An interactive version of this figure may be viewed at https://genome-note-higlass.tol.sanger.ac.uk/l/?d=b1h_oRM_RIu7RhwFE-mCfA.

**Table 2.** Chromosomal pseudomolecules in the genome assembly of *Hemithea aestivaria*, ilHemAest2.

INSDC accession	Chromosome	Length (Mb)	GC%
OX382324.1	1	20.12	36.5
OX382325.1	2	19.94	36.0
OX382326.1	3	19.83	36.5
OX382327.1	4	19.27	36.5
OX382328.1	5	19.24	36.5
OX382329.1	6	18.77	36.5
OX382330.1	7	18.76	36.0
OX382331.1	8	18.71	36.5
OX382332.1	9	18.48	36.0
OX382333.1	10	18.39	36.5
OX382334.1	11	17.79	36.5
OX382335.1	12	17.77	36.5
OX382336.1	13	17.63	36.5
OX382337.1	14	17.45	36.5
OX382338.1	15	16.89	36.5
OX382339.1	16	16.47	36.5
OX382340.1	17	16.46	36.5
OX382341.1	18	16.28	36.5
OX382342.1	19	16.08	36.5
OX382343.1	20	15.97	37.0
OX382344.1	21	15.65	37.0
OX382345.1	22	13.58	36.5
OX382346.1	23	12.88	36.5
OX382347.1	24	12.66	37.0
OX382348.1	25	11.92	36.5
OX382349.1	26	11.81	36.5
OX382350.1	27	10.84	37.5
OX382351.1	28	9.26	37.0
OX382352.1	29	8.3	37.0
OX382353.1	30	7.65	37.5
OX382323.1	Z	23.61	36.5
OX382354.1	MT	0.02	18.0

The estimated Quality Value (QV) of the final assembly is 59.5 with *k*-mer completeness of 100%, and the assembly has a BUSCO v5.3.2 completeness of 98.0% (single = 97.4%, duplicated = 0.6%), using the lepidoptera_odb10 reference set ( *n* = 5,286).

Metadata for specimens, spectral estimates, sequencing runs, contaminants and pre-curation assembly statistics can be found at https://links.tol.sanger.ac.uk/species/572857.

## Genome annotation report

The *Hemithea aestivaria* genome assembly (GCA_947507615.1) was annotated using the Ensembl rapid annotation pipeline ( Table 1; https://rapid.ensembl.org/Hemithea_aestivaria_GCA_947507615.1/Info/Index). The resulting annotation includes 18,682 transcribed mRNAs from 18,477 protein-coding genes.

## Methods

### Sample acquisition and nucleic acid extraction

The specimen selected for genome sequencing was a male *Hemithea aestivaria* (ilHemAest2), collected from Wytham Woods, Oxfordshire (biological vice-county Berkshire), UK (latitude 51.77, longitude –1.34) on 2020-07-05. The specimen was taken from woodland habitat by Douglas Boyes (University of Oxford) using a light trap. The specimen was identified by the collector, and then snap-frozen on dry ice.

DNA was extracted at the Tree of Life laboratory, Wellcome Sanger Institute (WSI). The ilHemAest2 sample was weighed and dissected on dry ice with tissue set aside for Hi-C sequencing. Whole organism tissue was disrupted using a Nippi Powermasher fitted with a BioMasher pestle. High molecular weight (HMW) DNA was extracted using the Qiagen MagAttract HMW DNA extraction kit. HMW DNA was sheared into an average fragment size of 12–20 kb in a Megaruptor 3 system with speed setting 30. Sheared DNA was purified by solid-phase reversible immobilisation using AMPure PB beads with a 1.8X ratio of beads to sample to remove the shorter fragments and concentrate the DNA sample. The concentration of the sheared and purified DNA was assessed using a Nanodrop spectrophotometer and Qubit Fluorometer and Qubit dsDNA High Sensitivity Assay kit. Fragment size distribution was evaluated by running the sample on the FemtoPulse system.

### Sequencing

Pacific Biosciences HiFi circular consensus d DNA sequencing libraries were constructed according to the manufacturers’ instructions. Poly(A) RNA-Seq libraries were constructed using the NEB Ultra II RNA Library Prep kit. DNA and RNA sequencing was performed by the Scientific Operations core at the WSI on the Pacific Biosciences SEQUEL II (HiFi) instrument. Hi-C data were also generated from tissue of ilHemAest2 that had been set aside, using the Arima2 kit and sequenced on the Illumina NovaSeq 6000 instrument.

### Genome assembly, curation and evaluation

Assembly was carried out with Hifiasm ( Cheng *et al.*, 2021) and haplotypic duplication was identified and removed with purge_dups ( Guan *et al.*, 2020). The assembly was then scaffolded with Hi-C data ( Rao *et al.*, 2014) using YaHS ( Zhou *et al.*, 2023). The assembly was checked for contamination and corrected described previously ( Howe *et al.*, 2021). Manual curation was performed using HiGlass ( Kerpedjiev *et al.*, 2018) and Pretext ( Harry, 2022). The mitochondrial genome was assembled using MitoHiFi ( Uliano-Silva *et al.*, 2022), which runs MitoFinder ( Allio *et al.*, 2020) or MITOS ( Bernt *et al.*, 2013) and uses these annotations to select the final mitochondrial contig and to ensure the general quality of the sequence.

A Hi-C map for the final assembly was produced using bwa-mem2 ( Vasimuddin *et al.*, 2019) in the Cooler file format ( Abdennur & Mirny, 2020). To assess the assembly metrics, the *k*-mer completeness and QV consensus quality values were calculated in Merqury ( Rhie *et al.*, 2020). This work was done using Nextflow ( Di Tommaso *et al.*, 2017) DSL2 pipelines “sanger-tol/readmapping” ( Surana *et al.*, 2023a) and “sanger-tol/genomenote” ( Surana *et al.*, 2023b). The genome was analysed within the BlobToolKit environment ( Challis *et al.*, 2020) and BUSCO scores ( Manni *et al.*, 2021; Simão *et al.*, 2015) were calculated.

Table 3 contains a list of relevant software tool versions and sources.

**Table 3.** Software tools: versions and sources.

Software tool	Version	Source
BlobToolKit	4.0.7	https://github.com/blobtoolkit/blobtoolkit
BUSCO	5.3.2	https://gitlab.com/ezlab/busco
Hifiasm	0.16.1-r375	https://github.com/chhylp123/hifiasm
HiGlass	1.11.6	https://github.com/higlass/higlass
Merqury	MerquryFK	https://github.com/thegenemyers/MERQURY.FK
MitoHiFi	2	https://github.com/marcelauliano/MitoHiFi
PretextView	0.2	https://github.com/wtsi-hpag/PretextView
purge_dups	1.2.3	https://github.com/dfguan/purge_dups
sanger-tol/genomenote	v1.0	https://github.com/sanger-tol/genomenote
sanger-tol/readmapping	1.1.0	https://github.com/sanger-tol/readmapping/tree/1.1.0
YaHS	yahs-1.1.91eebc2	https://github.com/c-zhou/yahs

### Genome annotation

The BRAKER2 pipeline ( Brůna *et al.*, 2021) was used in the default protein mode to generate annotation for the *Hemithea aestivaria* assembly (GCA_947507615.1) in Ensembl Rapid Release.

### Wellcome Sanger Institute – Legal and Governance

The materials that have contributed to this genome note have been supplied by a Darwin Tree of Life Partner. The submission of materials by a Darwin Tree of Life Partner is subject to the **‘Darwin Tree of Life Project Sampling Code of Practice’**, which can be found in full on the Darwin Tree of Life website here. By agreeing with and signing up to the Sampling Code of Practice, the Darwin Tree of Life Partner agrees they will meet the legal and ethical requirements and standards set out within this document in respect of all samples acquired for, and supplied to, the Darwin Tree of Life Project.

Further, the Wellcome Sanger Institute employs a process whereby due diligence is carried out proportionate to the nature of the materials themselves, and the circumstances under which they have been/are to be collected and provided for use. The purpose of this is to address and mitigate any potential legal and/or ethical implications of receipt and use of the materials as part of the research project, and to ensure that in doing so we align with best practice wherever possible. The overarching areas of consideration are:

- Ethical review of provenance and sourcing of the material
- Legality of collection, transfer and use (national and international)

Each transfer of samples is further undertaken according to a Research Collaboration Agreement or Material Transfer Agreement entered into by the Darwin Tree of Life Partner, Genome Research Limited (operating as the Wellcome Sanger Institute), and in some circumstances other Darwin Tree of Life collaborators.

## Data Availability

European Nucleotide Archive: *Hemithea aestivaria* (common emerald). Accession number PRJEB56491; https://identifiers.org/ena.embl/PRJEB56491. ( Wellcome Sanger Institute, 2022) The genome sequence is released openly for reuse. The *Hemithea aestivaria* genome sequencing initiative is part of the Darwin Tree of Life (DToL) project. All raw sequence data and the assembly have been deposited in INSDC databases. Raw data and assembly accession identifiers are reported in Table 1.

## Author information

Members of the University of Oxford and Wytham Woods Genome Acquisition Lab are listed here: https://doi.org/10.5281/zenodo.4789928.

Members of the Darwin Tree of Life Barcoding collective are listed here: https://doi.org/10.5281/zenodo.4893703.

Members of the Wellcome Sanger Institute Tree of Life programme are listed here: https://doi.org/10.5281/zenodo.4783585.

Members of Wellcome Sanger Institute Scientific Operations: DNA Pipelines collective are listed here: https://doi.org/10.5281/zenodo.4790455.

Members of the Tree of Life Core Informatics collective are listed here: https://doi.org/10.5281/zenodo.5013541.

Members of the Darwin Tree of Life Consortium are listed here: https://doi.org/10.5281/zenodo.4783558.

## References

[ref-1] Abdennur N Mirny LA : Cooler: Scalable storage for Hi-C data and other genomically labeled arrays. *Bioinformatics.* 2020;36(1):311–316. 10.1093/bioinformatics/btz540 31290943 PMC8205516

[ref-2] Allio R Schomaker-Bastos A Romiguier J : MitoFinder: Efficient automated large‐scale extraction of mitogenomic data in target enrichment phylogenomics. *Mol Ecol Resour.* 2020;20(4):892–905. 10.1111/1755-0998.13160 32243090 PMC7497042

[ref-3] Ban X Jiang N Cheng R : Tribal classification and phylogeny of Geometrinae (Lepidoptera: Geometridae) inferred from seven gene regions. *Zool J Linn Soc.* 2018;184(3):653–672. 10.1093/zoolinnean/zly013

[ref-4] Bernt M Donath A Jühling F : MITOS: Improved *de novo* metazoan mitochondrial genome annotation. *Mol Phylogenet Evol.* 2013;69(2):313–319. 10.1016/j.ympev.2012.08.023 22982435

[ref-5] Brůna T Hoff KJ Lomsadze A : BRAKER2: Automatic eukaryotic genome annotation with GeneMark-EP+ and AUGUSTUS supported by a protein database. *NAR Genom Bioinform.* 2021;3(1):lqaa108. 10.1093/nargab/lqaa108 33575650 PMC7787252

[ref-6] Challis R Richards E Rajan J : BlobToolKit – interactive quality assessment of genome assemblies. *G3 (Bethesda).* 2020;10(4):1361–1374. 10.1534/g3.119.400908 32071071 PMC7144090

[ref-7] Cheng H Concepcion GT Feng X : Haplotype-resolved de novo assembly using phased assembly graphs with hifiasm. *Nat Methods.* 2021;18(2):170–175. 10.1038/s41592-020-01056-5 33526886 PMC7961889

[ref-8] Cook M : The systematics of Emerald moths (Geometridae, Geometrinae). University of Oxford. 1993; (Accessed: 23 May 2023). Reference Source

[ref-9] Cook MA Harwood LM Scoble MJ : The chemistry and systematic importance of the green wing pigment in emerald moths (Lepidopera: Geometridae, Geometrinae). *Biochem Syst Ecol.* 1994;22(1):43–51. (Accessed: 23 May 2023). 10.1016/0305-1978(94)90113-9

[ref-16] Di Tommaso P Chatzou M Floden EW : Nextflow enables reproducible computational workflows. *Nat Biotechnol.* 2017;35(4):316–319. 10.1038/nbt.3820 28398311

[ref-10] Doǧanlar M Beirne BP : *Hemithea aestivaria*, a geometrid new to North America, established in British Columbia (Lepidoptera: Geometridae: Geometrinae). *Can Entomol.* 1979;111(10):1121–1121. 10.4039/Ent1111121a-10

[ref-11] GBIF Secretariat: *Hemithea aestivaria* (Hübner, 1799). *GBIF Backbone Taxonomy*. 2022; (Accessed: 3 March 2023). Reference Source

[ref-12] Guan D McCarthy SA Wood J : Identifying and removing haplotypic duplication in primary genome assemblies. *Bioinformatics.* 2020;36(9):2896–2898. 10.1093/bioinformatics/btaa025 31971576 PMC7203741

[ref-13] Harry E : PretextView (Paired REad TEXTure Viewer): A desktop application for viewing pretext contact maps. 2022; (Accessed: 19 October 2022). Reference Source

[ref-14] Hausmann A : Geometrid moths of Europe.Vol. 1: Introduction to the series. Archiearinae, Oenochrominae, Geometrinae. Stenstrup: Apollo Books. 2001; (Accessed: 19 October 2022).

[ref-15] Howe K Chow W Collins J : Significantly improving the quality of genome assemblies through curation. *GigaScience.* Oxford University Press. 2021;10(1):giaa153. 10.1093/gigascience/giaa153 33420778 PMC7794651

[ref-17] Kerpedjiev P Abdennur N Lekschas F : HiGlass: web-based visual exploration and analysis of genome interaction maps. *Genome Biol.* 2018;19(1):125. 10.1186/s13059-018-1486-1 30143029 PMC6109259

[ref-18] LaGasa E : Exotic fruit tree pests in Whatcom County, Washington.1996; Accessed: 3 March 2023). Reference Source

[ref-19] Looney C Murray T LaGasa E : Shadow Surveys: How Non-Target Identifications and Citizen Outreach Enhance Exotic Pest Detection. *Am Entomol.* 2016;62(4):247–254. 10.1093/ae/tmw063

[ref-20] Manni M Berkeley MR Seppey M : BUSCO Update: Novel and Streamlined Workflows along with Broader and Deeper Phylogenetic Coverage for Scoring of Eukaryotic, Prokaryotic, and Viral Genomes. *Mol Biol Evol.* 2021;38(10):4647–4654. 10.1093/molbev/msab199 34320186 PMC8476166

[ref-21] Randle Z Evans-Hill LJ Parsons MS : Atlas of Britain & Ireland’s Larger Moths. Newbury: NatureBureau.2019. Reference Source

[ref-22] Rao SSP Huntley MH Durand NC : A 3D map of the human genome at kilobase resolution reveals principles of chromatin looping. *Cell.* 2014;159(7):1665–1680. 10.1016/j.cell.2014.11.021 25497547 PMC5635824

[ref-24] Rhie A McCarthy SA Fedrigo O : Towards complete and error-free genome assemblies of all vertebrate species. *Nature.* 2021;592(7856):737–746. 10.1038/s41586-021-03451-0 33911273 PMC8081667

[ref-23] Rhie A Walenz BP Koren S : Merqury: Reference-free quality, completeness, and phasing assessment for genome assemblies. *Genome Biol.* 2020;21(1):245. 10.1186/s13059-020-02134-9 32928274 PMC7488777

[ref-25] Schmidt C Anctil A : *Hemithea aestivaria* (Hübner) (Lepidoptera: Geometridae), a Palaearctic moth, new to eastern North America. *Biodivers Data J.* 2021;9:e64985. 10.3897/BDJ.9.e64985 34084069 PMC8163713

[ref-26] Sihvonen P Mutanen M Kaila L : Comprehensive Molecular Sampling Yields a Robust Phylogeny for Geometrid Moths (Lepidoptera: Geometridae). *PLoS One.* 2011;6(6):e20356. 10.1371/journal.pone.0020356 21673814 PMC3106010

[ref-27] Simão FA Waterhouse RM Ioannidis P : BUSCO: assessing genome assembly and annotation completeness with single-copy orthologs. *Bioinformatics.* 2015;31(19):3210–3212. 10.1093/bioinformatics/btv351 26059717

[ref-28] South R : Moths of the British Isles. New edition. London: Frederick Warne and Co.1961.

[ref-30] Surana P Muffato M Qi G : sanger-tol/readmapping: sanger-tol/readmapping v1.1.0 - Hebridean Black (1.1.0). *Zenodo* .2023a; (Accessed: 17 April 2023). 10.5281/zenodo.7755665

[ref-29] Surana P Muffato M Sadasivan Baby C : sanger-tol/genomenote (v1.0.dev). *Zenodo* .2023b; (Accessed: 17 April 2023). 10.5281/zenodo.6785935

[ref-31] Uliano-Silva M Ferreira JGRN Krasheninnikova K : MitoHiFi: a python pipeline for mitochondrial genome assembly from PacBio High Fidelity reads. *bioRxiv.* [Preprint].2022. 10.1101/2022.12.23.521667 PMC10354987 37464285

[ref-32] Vasimuddin Md Misra S Li H : Efficient Architecture-Aware Acceleration of BWA-MEM for Multicore Systems. In: *2019 IEEE International Parallel and Distributed Processing Symposium (IPDPS).*IEEE,2019;314–324. 10.1109/IPDPS.2019.00041

[ref-33] Wellcome Sanger Institute: The genome sequence of the Common Emerald, *Hemithea aestivaria* (Hübner, 1789), European Nucleotide Archive. [dataset], accession number PRJEB56491.2022.

[ref-34] Zhou C McCarthy SA Durbin R : YaHS: yet another Hi-C scaffolding tool. *Bioinformatics.* Edited by C. Alkan,2023;39(1):btac808. 10.1093/bioinformatics/btac808 36525368 PMC9848053

