# Supplementary material for: The genome sequence of the Common Emerald, Hemithea aestivaria (Hübner, 1789)
Source: Wellcome Open Res. 2023 Jun 14;8:243. [Version 1] doi: 10.12688/wellcomeopenres.19542.1 (PMC11099506; doi:10.12688/wellcomeopenres.19542.1)
Supplement: Peer review history

# Reviewer report: Reviewer response for version 1

**DOI:** 10.21956/wellcomeopenres.21647.r76292

**Published:** 2024-05-16

**Authors:** Manuela Lopez Villavicencio

**Affiliations:** 1 Muséum National d'Histoire Naturelle, Paris, France

This article reports the genome assembly of a male individual of the Common Emerald, *Hemithea aestivaria*. The assembly included the 30 autosomes, the Z sexual chromosome and the mitochondrial genome. Besides the assembly, the authors performed the annotation of the genome.

 The article is clearly written and interesting to read. It shows convincing evidences for a high-quality assembly of the genome based on BUSCO scores. The methods for genome assembly, quality test and Hi-C scaffolding are relevant and up-to-date. There are sufficient details of methods and materials provided to allow replication by others.

 The assembly sequence was assigned to 31 chromosomal-level scaffolds, representing 30 autosomes plus the sexual Z chromosome. The annotation identified more than 18,000 coding genes for this species.  

 As I am interested in butterfly genomes, I would have loved to have other important pieces of information such as the genome-wide level of heterozygosity (estimated with Jellyfish and GenomeScope) or the percentage of repeated elements in the genome, which the authors obtained during the softmasking step prior to annotation. Additionally, I believe it is important for the authors to provide a BUSCO quality assessment for the annotation.

 Overall, I think the release of this well-assembled and annotated genome is a very useful contribution and I recommend the approval of this article.

- Are sufficient details of methods and materials provided to allow replication by others?

Yes, but please include BUSCO scores for annotation

Are sufficient details of methods and materials provided to allow replication by others?

Yes

Is the rationale for creating the dataset(s) clearly described?

Yes

Are the datasets clearly presented in a useable and accessible format?

Yes

Are the protocols appropriate and is the work technically sound?

Yes

Reviewer Expertise:

Evolutionary biology

I confirm that I have read this submission and believe that I have an appropriate level of expertise to confirm that it is of an acceptable scientific standard.

# Reviewer report: Reviewer response for version 1

**DOI:** 10.21956/wellcomeopenres.21647.r76298

**Published:** 2024-05-11

**Authors:** Arun Arumugaperumal

**Affiliations:** 1 Department of Biotechnology, Rajalakshmi Engineering College, Chennai, Tamilnadu, India

This is the first report of the genome sequence of the common emerald Hemithea aestivaria. The genome is of size 500 Mbp and has 31 chromosomes. The authors have predicted 18,477 genes in the genome. A 17 kbp mitochondrial genome was also assembled. The genome sequence was shown to be 98% complete through BUSCO analysis. The genome can be accessed from NCBI using the identifier GCA_947507615.1. 

 The photograph of the specimen could have been taken with a standard background along with a scale. The authors could have predicted the non-coding RNA genes and provided the data also.

Are sufficient details of methods and materials provided to allow replication by others?

Yes

Is the rationale for creating the dataset(s) clearly described?

Yes

Are the datasets clearly presented in a useable and accessible format?

Yes

Are the protocols appropriate and is the work technically sound?

Yes

Reviewer Expertise:

genomics, bioinformatics

I confirm that I have read this submission and believe that I have an appropriate level of expertise to confirm that it is of an acceptable scientific standard.
